# Wearable Soft Sensors for Spinal Monitoring: Soft Materials, Multimodal Integration, and Clinical Translation

**DOI:** 10.3390/mi17101210

**Published:** 2026-10-21

**Authors:** Chengyu Li, Shuxing Xu, Wenbo Li, Dian Jin, Yudong Zhao, Chao Zhang, Weishi Li, Zhuoran Sun, Siyu Zhou

**Affiliations:** 1 Department of Orthopaedics, Peking University Third Hospital, Beijing 100191, China; 2 School of Fashion and Textiles, the Hong Kong Polytechnic University, Hong Kong 999077, China; lichengyu2018@163.com (C.L.); wenboo.li@polyu.edu.hk (W.L.);; 3 Beijing WeiNa ZhiLian Technology Co., Ltd., Beijing 101101, China; xushuxing@binn.cas.cn; 4 School of Nanoscience and Engineering, University of Chinese Academy of Sciences, Beijing 100049, China; 5 Engineering Research Center of Bone and Joint Precision Medicine, Ministry of Education, Beijing 100191, China; 6 School of Basic Medical Sciences, Peking University Health Science Center, Beijing 100191, China

**Keywords:** soft sensors, spinal monitoring, flexible materials, signal processing, clinical applications

## Abstract

Spinal musculoskeletal disorders demand continuous assessment of movement and loading, whereas routine clinical imaging provides important but episodic structural information. While wearable soft sensors offer a non-invasive approach to complement these assessments, translating laboratory-scale prototypes into robust clinical systems remains challenging because of electromechanical stability, signal fidelity, and data interpretation. This review critically examines the end-to-end development of soft wearable technologies for spinal monitoring, moving beyond a materials-focused survey to address these translational gaps. First, we evaluate advances in soft sensing mechanisms, highlighting the trade-offs between mechanical compliance and stable sensor performance. We then examine system-level integration, including soft or flexible sensing elements, conventional sensors on compliant substrates, rigid–soft interfaces, and multimodal systems for monitoring posture, motion, pressure, and muscle activity. Furthermore, we discuss signal-processing and artificial intelligence-assisted workflows for denoising and extracting meaningful physiological features. By comparing reported performance metrics and validation settings, we assess representative applications in scoliosis and chronic low back pain. Current evidence demonstrates technical feasibility in laboratory tests and small studies, but does not yet establish broad diagnostic benefit or routine clinical effectiveness. Finally, we identify key evidence gaps, including external validation, patient-based studies, standardized protocols, and long-term assessment, that must be addressed for clinical translation.

## 1. Introduction

As the core structure for human movement and support, the health status of the spine directly impacts physical function and quality of life. Scoliosis and chronic low back pain (LBP) are currently highly prevalent spinal diseases worldwide, emerging as significant public health issues [1,2]. Adolescent idiopathic scoliosis is the most common musculoskeletal deformity in adolescents [3,4]. Without timely intervention, it can lead to severe spinal deformity and impaired cardiopulmonary function. Meanwhile, chronic LBP affects individuals across all age groups and represents one of the leading causes of physical dysfunction and reduced quality of life, imposing a sustained burden on healthcare systems. The onset and progression of such spinal diseases are closely associated with long-term poor posture and abnormal dynamic spinal loading. Therefore, early monitoring, precise assessment, and continuous rehabilitation tracking are crucial for effective disease management.

Traditional diagnosis and rehabilitation monitoring of spinal diseases rely heavily on imaging technologies such as X-ray, computed tomography (CT), and magnetic resonance imaging (MRI) [1,2]. Although these modalities can provide high-resolution static imaging of spinal anatomical structures to facilitate diagnosis, they possess core limitations that cannot be ignored. First, radiographic examinations entail the risk of ionizing radiation, rendering them unsuitable for long-term continuous monitoring [5]. Repeated testing poses specific health hazards, particularly for the adolescent population. Second, they merely capture the static state of the spine at a specific time point, failing to record dynamic movements, load variations, and postural habits during activities of daily living. Thus, they struggle to reflect the true progression of the disease and the efficacy of rehabilitation. Third, these examinations must be conducted in specialized clinical settings, involving cumbersome procedures and high costs. The lack of convenient, home-based monitoring methods leads to a severe deficiency in out-of-hospital tracking during the rehabilitation phase, causing a significant disconnect between clinical treatment and home rehabilitation. Furthermore, traditional rehabilitation assessments largely depend on physicians’ subjective observations and patients’ self-reports. The absence of objective, quantitative monitoring indicators hampers the formulation of personalized rehabilitation protocols and the evaluation of their effectiveness.

The rapid development of wearable flexible sensing technology provides a novel solution to overcome these limitations, becoming a prominent research hotspot in the field of spinal health monitoring [6]. Compared to traditional rigid sensing devices, wearable flexible sensors are fundamentally based on soft and stretchable materials. They exhibit excellent skin conformability and dynamic adaptability, enabling non-invasive, continuous, quantitative, and home-based monitoring of spinal health, thereby bridging the gap between clinical static diagnosis and out-of-hospital dynamic monitoring. This technology can capture multi-dimensional data in real-time, such as spinal angle variations, dynamic loads, and surrounding muscle electromyographic (EMG) signals [7,8,9]. It provides objective and quantitative evidence for early screening, severity assessment, postoperative rehabilitation, and daily prevention of spinal diseases. Simultaneously, it allows for the prompt correction of poor posture via real-time feedback, driving a paradigm shift in spinal disease management from passive treatment to proactive prevention.

This article presents a technology-focused review of wearable sensing systems relevant to spinal monitoring, covering developments from material design, sensor integration, and system construction to signal processing and clinical applications. Unlike a systematic review, this work does not employ a prespecified database search, duplicate screening, formal risk-of-bias assessment, or PRISMA flow process, and therefore does not claim exhaustive coverage. Instead, studies are selected for their relevance to sensing mechanisms, wearable integration, signal processing, and spinal-health applications. The review covers three major categories of sensing technologies: (i) intrinsically soft or flexible sensing elements, (ii) conventional sensing components integrated into compliant textiles or substrates, and (iii) multimodal systems combining soft and rigid components. Here, soft sensor refers specifically to a sensing element whose active material or device structure is mechanically compliant, whereas a soft wearable system may additionally incorporate rigid inertial measurement units (IMUs), electronics, or surface electromyography (sEMG) modules. This distinction is applied throughout the following comparisons. First, we elaborate on the soft and hybrid material systems used for spinal monitoring, including piezoresistive/strain-sensitive materials, flexible triboelectric and piezoelectric materials, fabric- and fiber-based substrates, and rigid–soft composite strategies. We then analyze wearable integration strategies for flexible and elastic sensors, comparing reported sensing performance and discussing the advantages and limitations of multimodal fusion. Subsequently, we introduce the overall design and wearing configurations of wearable spinal-monitoring systems, encompassing hardware architecture, wireless transmission, and adaptability to different monitoring scenarios. We further examine signal-processing workflows and intelligent algorithms, including signal denoising, posture and motion estimation, artificial intelligence (AI)-based classification, and digital biomarker extraction. Finally, we assess application evidence in representative scenarios such as scoliosis, chronic LBP, and postoperative rehabilitation, while considering the validation settings and study-specific performance metrics reported in the literature. Although current studies demonstrate technical feasibility across laboratory tests and small human studies, broader diagnostic benefit and routine clinical effectiveness remain insufficiently established. Accordingly, we identify key translational challenges, including long-term stability, external validation, patient-based studies, standardized protocols, clinical adaptability, and long-term assessment. Addressing these gaps will be essential for advancing wearable flexible sensing from technological development toward reliable clinical integration and supporting the broader management of spinal health.

## 2. Soft and Hybrid Materials for Spinal Wearable Sensing

Conventional approaches for spinal health assessment rely predominantly on imaging modalities such as X-ray, computed tomography, and magnetic resonance imaging. Although these techniques provide high spatial resolution, they suffer from several intrinsic limitations, including radiation exposure, high cost, and the inability to capture continuous biomechanical dynamics. Moreover, these methods are typically confined to clinical settings, rendering them unsuitable for long-term monitoring of spinal posture and rehabilitation progress. Emerging wearable sensing technologies offer a promising alternative by enabling continuous, non-invasive, and quantitative monitoring in daily environments [10,11]. However, the rigid nature of traditional electronic systems introduces mechanical mismatch with soft biological tissues, leading to discomfort, motion artefacts, and reduced compliance during prolonged use. These challenges have driven increasing interest in soft and hybrid material systems that can conform intimately to the human body while maintaining high sensing performance.

**Figure 1:**
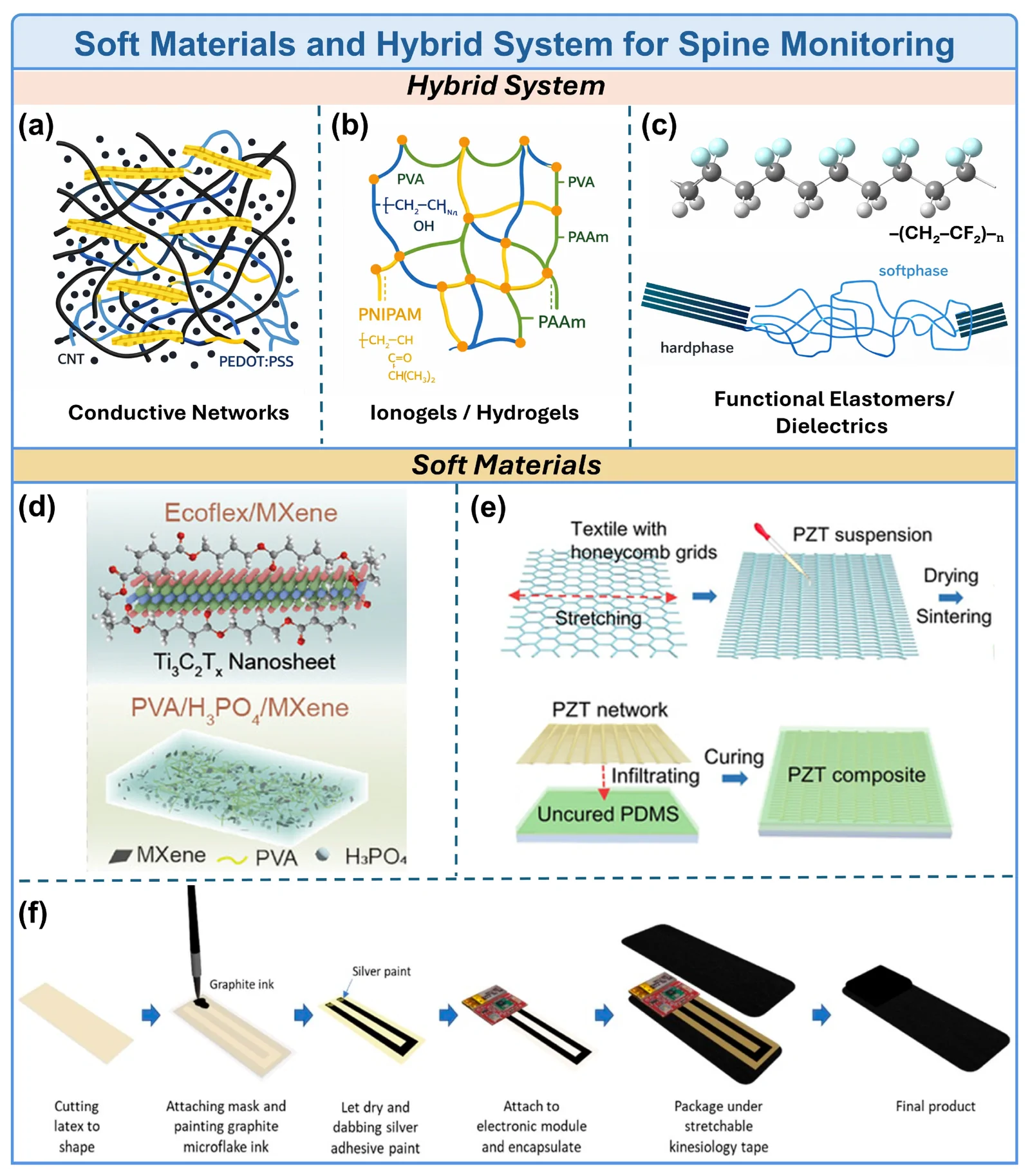
Soft materials and hybrid system for spine monitoring. (**a**) The conductive networks formed by carbon-based nanomaterials or conductive polymers enable piezoresistive sensing. (**b**) Representative hydrogel networks composed of polymer chains such as PVA, PAAm and PNIPAM. (**c**) Functional polymer systems, including fluoropolymer-based piezoelectric materials and elastomeric networks. (**d**) Representative hybrid material systems for self-powered and high-performance wearable sensing (reprinted from Ref. [14], Copyright 2026, with permission from Wiley-VCH GmbH). (**e**) Structure-engineered piezoelectric materials for flexible and anisotropic sensing (reprinted from Ref. [15], Copyright 2021, with permission from AAAS). (**f**) Fully integrated wearable sensing systems combining soft materials, textiles and electronic modules, enabling practical deployment for continuous spine monitoring (reprinted from Ref. [18], Copyright 2020, with permission by CC BY 4.0).

Soft materials, characterized by low elastic modulus and high deformability, are particularly well suited for interfacing with skin and musculoskeletal structures. Among them, piezoresistive strain-sensitive materials represent one of the most widely explored approaches for spinal motion monitoring. These systems typically rely on conductive networks formed by carbon-based nanomaterials such as carbon nanotubes or conductive polymers embedded within elastomeric matrices (Figure 1a). Upon mechanical deformation, the conductive pathways undergo reversible disruption or reconstruction, leading to measurable resistance changes. In these systems, network rearrangement, tunnelling distance, and contact resistance collectively determine the sensing response. For example, conductive elastomer-based e-textile systems integrated with stretchable fabrics have been developed to monitor lumbar spine curvature by tracking distributed strain along the back [12]. In such systems, multiple sensing units are strategically positioned along the spine, and their signals are further combined with inertial measurement units to reconstruct spinal posture with improved accuracy. In a bionic MXene/sponge sensor, dual conductive pathways yielded reported pressure sensitivities of 1.3 × 10^6^ kPa^−1^ in the low-pressure regime and 1.9 × 10^5^ kPa^−1^ in the higher-pressure regime; an eight-channel dorsal array classified predefined sitting postures with 100% accuracy in the reported experimental dataset. These values are device- and dataset-specific and should not be interpreted as general performance benchmarks for piezoresistive sensing. Despite their structural simplicity and high sensitivity, these piezoresistive systems often suffer from hysteresis, signal drift, and environmental instability, which may compromise long-term reliability.

Conductive hydrogels have emerged as another important class of soft sensing materials due to their intrinsic softness, high water content, and ionic conductivity. Hydrogels can conform intimately to skin and maintain stable electrical performance under large deformation, making them suitable for long-term wearable applications (Figure 1b). Their electrical conduction is mediated by mobile ions within the water-rich polymer network; consequently, conductivity and electrode–skin impedance depend on ion concentration and mobility, water retention, polymer crosslinking, and contact area. In recent work, in situ gelation strategies have been employed to form hydrogel-based electromyography sensors directly on the skin, significantly improving electrode–skin contact and signal fidelity [13]. In a gelatin/sodium pyrrolidone carboxylate (PCA-Na) in-situ-gelling hydrogel EMG interface, incorporation of PCA-Na improved ionic transport: at 40 wt% PCA-Na, the resistance was reported to decrease by 82% relative to the gelatin hydrogel, while the complete electrode system exhibited an interfacial impedance of 40 kΩ/cm^2^ at 10 Hz and an EMG signal-to-noise ratio of 23.28 dB. These system-level values should not be interpreted as intrinsic bulk conductivity of the hydrogel. Increasing salt content can enhance ion transport but may simultaneously alter gelation, mechanical properties, adhesion, and water retention; therefore, conductivity should be evaluated together with impedance, hydration, mechanical properties, and signal quality. Unlike traditional strain sensors, such systems enable indirect assessment of spinal function by monitoring muscle activity associated with spinal stabilization. In addition, hydrogels have been widely used as stretchable electrodes in triboelectric nanogenerators, where poly(vinyl alcohol) (PVA)-based hydrogels enhance water retention and long-term stability [14]. These triboelectric systems convert mechanical motion into electrical signals through contact electrification and electrostatic induction, enabling self-powered monitoring of neck and spinal movements without continuously powering the sensing element (Figure 1d), although the associated readout and communication electronics still require energy.

Piezoelectric materials provide an alternative sensing mechanism based on electromechanical coupling. Fluoropolymer-based materials such as poly(vinylidene fluoride) and its copolymers exhibit strong piezoelectric responses and mechanical flexibility, enabling the detection of dynamic strain and vibration associated with spinal motion (Figure 1c). Their output is strongest under changing mechanical loads, making them particularly suitable for dynamic events, vibration, and transient deformation, whereas they generally provide less information about sustained static postures. Through rational structural design, including the development of piezoelectric ceramic-based fibers and composites, intrinsically brittle piezoelectric materials can be transformed into flexible and wearable formats, significantly expanding their applicability in conformal biointerfaces, as demonstrated in recent studies for spine monitoring (Figure 1e) [15]. Reported piezoelectric performance depends strongly on material phase, polarization conditions, device geometry, mechanical loading, and readout circuitry. Therefore, a single sensitivity value or universal sensitivity range cannot be assigned to the entire material class. Beyond wearable configurations, piezoelectric materials have also been integrated into implantable systems. For instance, metamaterial-inspired piezoelectric implants have been developed to monitor bone healing processes in spinal repair, where mechanical loading generates electrical signals that can be wirelessly detected [16]. Although these systems demonstrate high sensitivity and self-powered operation, their applicability to continuous posture monitoring remains limited due to reduced response under static deformation.

Textile-based substrates play a critical role as structural platforms for integrating soft sensing materials into wearable systems. Natural and synthetic fibers, including cotton, polyester, and elastomeric yarns, provide breathable, lightweight, and mechanically compliant interfaces with the human body. However, textile substrates themselves do not necessarily constitute intrinsically soft sensors; fibers and garments can instead host printed or embedded resistive traces, optical fibers, or even rigid electronic modules. Recent advances have demonstrated the seamless integration of polymer-based sensors into textile structures through direct printing or embedding techniques, enabling durable and comfortable wearable devices for motion monitoring (Figure 1f) [17]. For example, a knitted undergarment incorporating four silicone-embedded fiber Bragg grating (FBG) sensors was evaluated for force sensing under loads up to 10 N using an artificial-skin setup. This result represents a bench-level validation of garment–brace interface measurement rather than clinical patient monitoring. Textile structures can improve wearability and distribute sensing sites, but fit, laundering, slippage, and garment tension can substantially affect calibration and measurement stability. Furthermore, textile-based systems are often combined with other sensing modalities, such as inertial sensors, to improve robustness and reduce error accumulation in long-term measurements [18]. Accordingly, textile-mounted IMUs and sEMG electrodes are better regarded as components of a compliant wearable system rather than intrinsically soft sensing elements.

To further enhance sensing performance and functionality, hybrid material strategies that combine organic soft matrices with inorganic nanostructures have been extensively explored. Incorporation of functional fillers such as barium titanate nanoparticles, zinc oxide nanowires, conductive carbon nanomaterials, or other functional nanostructures into polymer matrices can establish percolating conductive pathways and enhance sensitivity through dielectric enhancement, interfacial polarization, tunnelling, or contact-resistance modulation. However, increasing filler concentration and improving percolation may also raise material stiffness, introduce hysteresis, and reduce mechanical durability. In addition, filler dispersion strongly affects the reproducibility of electrical pathways and therefore the sensing response. Anisotropic composite designs, where conductive or ionic pathways are directionally engineered, enable the differentiation of complex deformation modes, such as bending versus twisting, which is critical for accurate spinal motion analysis [19]. Beyond material composition, device architectures have also evolved toward higher integration density. Active-matrix sensing arrays, inspired by display technologies, have been introduced to achieve high-resolution spatial mapping of strain distribution across the lumbar region, enabling more precise reconstruction of spinal curvature [20]. Importantly, reported performance should always be interpreted in relation to the measured physical quantity, operating range, loading rate, device architecture, and comparator; metrics such as pressure sensitivity, angular error, displacement resolution, and classification accuracy are not directly interchangeable.

In parallel, multimodal and self-powered sensing systems have emerged as a major research direction. Triboelectric sensor arrays combined with deep learning algorithms have demonstrated the capability to decode complex motion patterns from distributed electrical signals, providing robust classification of spinal and cervical movements [14]. For example, a retractable triboelectric stretch sensor reported a sensitivity of 8 V mm^−1^, a minimum resolution of 0.6 mm, and more than 120,000 operational cycles in device-level tests. A separate stretchable triboelectric array reported approximately 1° angular resolution, an angle sensitivity of 0.56 V °^−1^, and 98.7% accuracy for classification of 14 predefined postures [14]. These metrics originate from different experiments and should not be interpreted as evidence of diagnostic accuracy in patients. Similarly, recent electronic skin systems integrate strain sensing with thermochromic responses, enabling simultaneous electrical and visual feedback of deformation [21]. These hybrid systems highlight the growing trend toward integrating multiple sensing mechanisms within a single platform to improve accuracy, robustness, and user interaction. Beyond wearable surface sensors, system-level integration has further expanded the scope of spinal monitoring technologies. Flexible electronic systems incorporating electromagnetic sensing, soft substrates, and advanced signal processing have achieved high-precision tracking of spinal implants and structural changes in vivo [11,20]. These systems demonstrate that the future of spinal monitoring lies not only in material innovation but also in the integration of sensing, data processing, and intelligent interpretation.

Overall, soft and hybrid material systems have enabled a transition from rigid, single-mode sensing toward conformal, multimodal platforms capable of capturing complex spinal biomechanics. Piezoresistive networks are readily patterned and can provide high sensitivity but remain vulnerable to hysteresis and drift; ionic hydrogels offer conformal bioelectrical contact but depend strongly on hydration and interface stability; triboelectric devices are attractive for dynamic, self-powered transduction but are sensitive to contact conditions; optical FBG systems are immune to electromagnetic interference but require an interrogator and careful mechanical coupling; and IMUs provide compact orientation information but can accumulate error and remain conventional rigid electronic components. Taken together, the literature indicates trade-offs rather than a single dominant sensing platform. The numerical values summarized in Table 1 should therefore be regarded as representative results from individual studies rather than head-to-head comparisons across material or device classes. Future developments will require improved long-term stability, standardized evaluation protocols and deeper integration with data-driven models to facilitate clinical translation.

## 3. Wearable Integration Strategies of Soft Sensors for Spinal Monitoring

**Figure 2:**
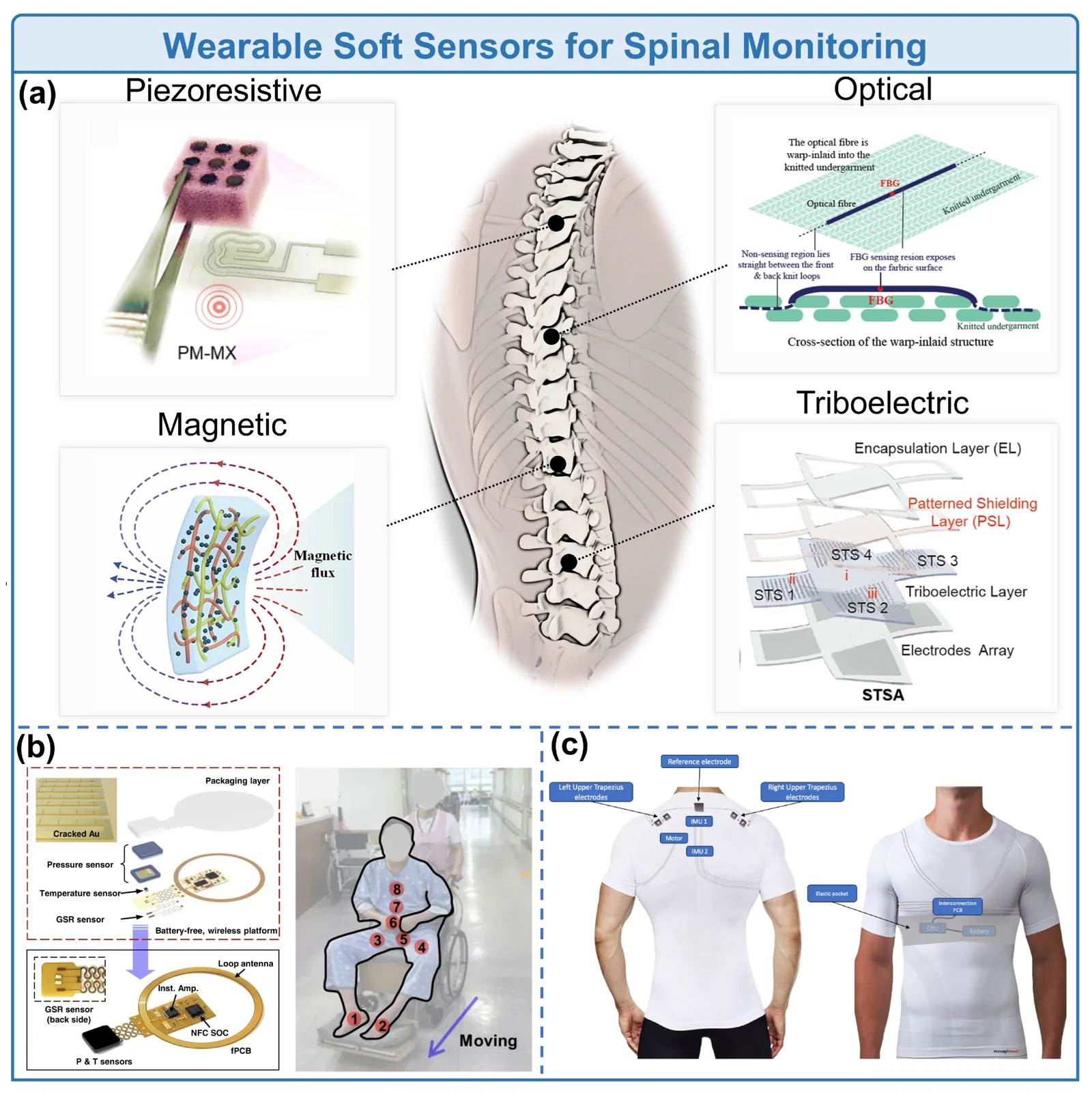
Wearable soft sensors for spinal monitoring. (**a**) Schematic illustration and representative prototypes of diverse soft sensing technologies for spinal monitoring, including piezoresistive, magnetic, optical and triboelectric sensors, showing the flexible deployment and structural design for spinal motion, stress and posture detection (reprinted from [22], Copyright 2024, with permission from Elsevier; reprinted from [11], Copyright 2024, with permission from Elsevier; reprinted from [23], Copyright 2023, with permission by CC BY 4.0; reprinted from [14], Copyright 2025, with permission from Wiley-VCH). (**b**) Wireless battery-free multimodal sensor for synchronous monitoring of spinal peripheral pressure, temperature and skin moisture (reprinted from [29], Copyright 2023, with permission by CC BY 4.0). (**c**) E-textile smart vest integrated with sEMG and IMU sensors for spinal kinematics and paraspinal muscle signal acquisition (reprinted from [30], Copyright 2024, with permission by CC BY 4.0).

The wearable integration technology of soft or flexible sensors for spinal monitoring represents a cutting-edge direction that breaks through the application limitations of traditional rigid sensing devices in spinal health monitoring. Although traditional rigid sensing enables accurate detection of spinal motion parameters, it fails to meet the practical demands of daily prevention and clinical rehabilitation due to poor wearing comfort, insufficient dynamic adaptability, motion artifact interference, and the inability to achieve long-term continuous monitoring. In contrast, advances in soft sensing technology have enabled technological evolution from single-point sensing to arrayed deployment and from single-parameter detection to multi-modal fusion through innovations in sensing principles, optimization of structural design, and upgrading of integration schemes, emerging as a core technical system for quantitative spinal monitoring. As illustrated in Figure 2a, typical soft sensing technologies such as piezoresistive, magnetic, optical, and triboelectric types are exemplified, intuitively demonstrating the diverse technical approaches of current soft sensing for spinal monitoring. For instance, biomimetic piezoresistive sensors with dual-conductive networks enable high-sensitivity monitoring under large spinal deformations [22]. When deployed in arrays, these sensors achieve global spinal behavioral capture and precise early warning for unhealthy postures. A reported bionic piezoresistive array achieved high pressure sensitivity and 100% recognition accuracy for its predefined sitting-posture classes in a controlled experimental dataset. However, this 100% value represents a classification result within the reported task and dataset rather than a general accuracy benchmark or evidence of clinical diagnostic accuracy. At the same time, a lightweight and low-power wearable monitoring system developed based on a high-sensitivity soft hydrogel strain sensor with an island–bridge configuration forms a complement to this integration scheme [27]. The island–bridge design facilitates mechanical deformation and conformability at the system level, although the electronic components located on the rigid islands may remain mechanically rigid. These examples collectively demonstrate that soft sensing should be distinguished from the broader concept of mechanically compliant wearable integration.

Furthermore, wireless, battery-free implantable magnetic sensing systems break the boundaries of surface monitoring [11], providing real-time internal monitoring of spinal motion with high structural reliability for long-term postoperative recovery. Based on the principles of optical measurement, Ka-Po Lee et al. proposed a textile-integrated sensing system by embedding fiber Bragg grating (FBG) optical fiber sensors into silica gel and integrating the composite module with knitted underwear [23]. This architecture provides a compliant textile platform for force measurement at a brace interface and demonstrates the potential of optical sensing for wearable spinal applications. In the reported bench protocol, the FBG smart textile was evaluated under loads up to 10 N using an artificial-skin setup. Thus, the reported results represent bench-level validation of garment–brace interface force measurement rather than direct evidence of patient-level spinal monitoring or clinical outcome improvement. In another notable study, Juan Tao et al. developed an array of self-powered stretchable triboelectric sensors combined with deep learning algorithms, achieving high-precision recognition of spinal postures and real-time corrective feedback [14]. The reported system achieved approximately 1° angular resolution, an angle sensitivity of 0.56 V °^−1^, and 98.7% classification accuracy across 14 predefined postures. Meanwhile, the arrayed design of carbon nanotube elastic fabric motion tape sensors designed by Elijah Wyckoff et al. further improves the signal stability and dimensional comprehensiveness of lumbar motion monitoring [28], which together with the triboelectric sensing array confirms that arrayation is a key strategy for adapting soft sensing to global spinal monitoring.

In addition, as shown in Figure 2b, the wireless multi-modal sensing system proposed by Seokjoo Cho et al. realizes synchronous and continuous monitoring of multiple parameters including pressure, temperature, and skin moisture [29]. Through batteryless design and nanoscale packaging optimization, it achieves integrated detection of physiological and mechanical parameters around the spine, providing multi-dimensional data support for the early warning of spinal pressure injuries in sedentary populations and also verifying the importance of multi-parameter fusion monitoring. Similarly, as depicted in Figure 2c, João Martins et al. integrated surface electromyography (sEMG) sensing and inertial measurement unit (IMU) inertial sensing into an electronic textile smart vest, realizing synchronous acquisition of spinal motion parameters and physiological signals of surrounding muscles [30]. The detection performance of its textile electrodes is superior to that of traditional commercial electrodes, and the accuracy of spinal angle estimation meets the requirements of clinical monitoring.

From single-point sensing to arrayed deployment, and from single-principle detection to multi-modal fusion, the wearable integration strategies of soft sensors for spinal monitoring have developed around the need to acquire spatially distributed and complementary biomechanical information. As summarized in Table 1, diverse sensing principles including IMU inertial sensing, flexible strain sensing, sEMG electromyographic sensing, and optical capture each exhibit distinctive technical merits and differentiated characteristics in core performance metrics such as detection accuracy, wearing comfort, and power consumption, enabling precise adaptation to specific clinical scenarios including scoliosis, chronic LBP, and postoperative spinal rehabilitation. These technologies can be adapted to different research and clinical scenarios, including scoliosis assessment, chronic low-back-pain-related motion analysis, posture monitoring, and postoperative spinal rehabilitation. However, Table 1 is intended to compare sensing mechanisms and representative study-level metrics rather than establish a ranking of technologies. Because the cited studies use different applied loads, sensor placements, reference instruments, participant populations, operating ranges, and datasets, their numerical values are not directly comparable. Where the available spinal-monitoring literature does not report a comparable operating range, accuracy metric, or validation protocol, this limitation should remain explicit rather than being inferred from results obtained under different conditions. Multi-modal fusion sensing, by integrating the advantages of different sensing technologies, realizes synchronous acquisition of multi-dimensional data including spinal motion, stress, muscle physiology, and skin parameters, making up for the detection limitations of single-sensing technology and becoming a core development trend of current soft sensing technology for spinal monitoring. Meanwhile, integrated designs featuring textile integration, lightweight construction, and low power consumption further enhance the home adaptability and long-term usability of wearable systems, laying a technical foundation for continuous quantitative monitoring of spinal health.

In the future, the wearable integration of soft sensors for spinal monitoring will further focus on the in-depth fusion of different sensing technologies, the collaborative upgrading of arrayation and intelligent analysis, and precise adaptation to clinical scenarios. Rather than relying solely on material-level improvements, the field will increasingly require coordinated optimization across sensing materials, device architectures, wearable integration, signal processing, and clinical validation. Such a system-level approach could facilitate the transition of soft and flexible sensing platforms from proof-of-concept laboratory demonstrations toward reliable long-term monitoring and clinically meaningful applications in spinal health.

## 4. System Design & Wearable Forms

**Figure 3:**
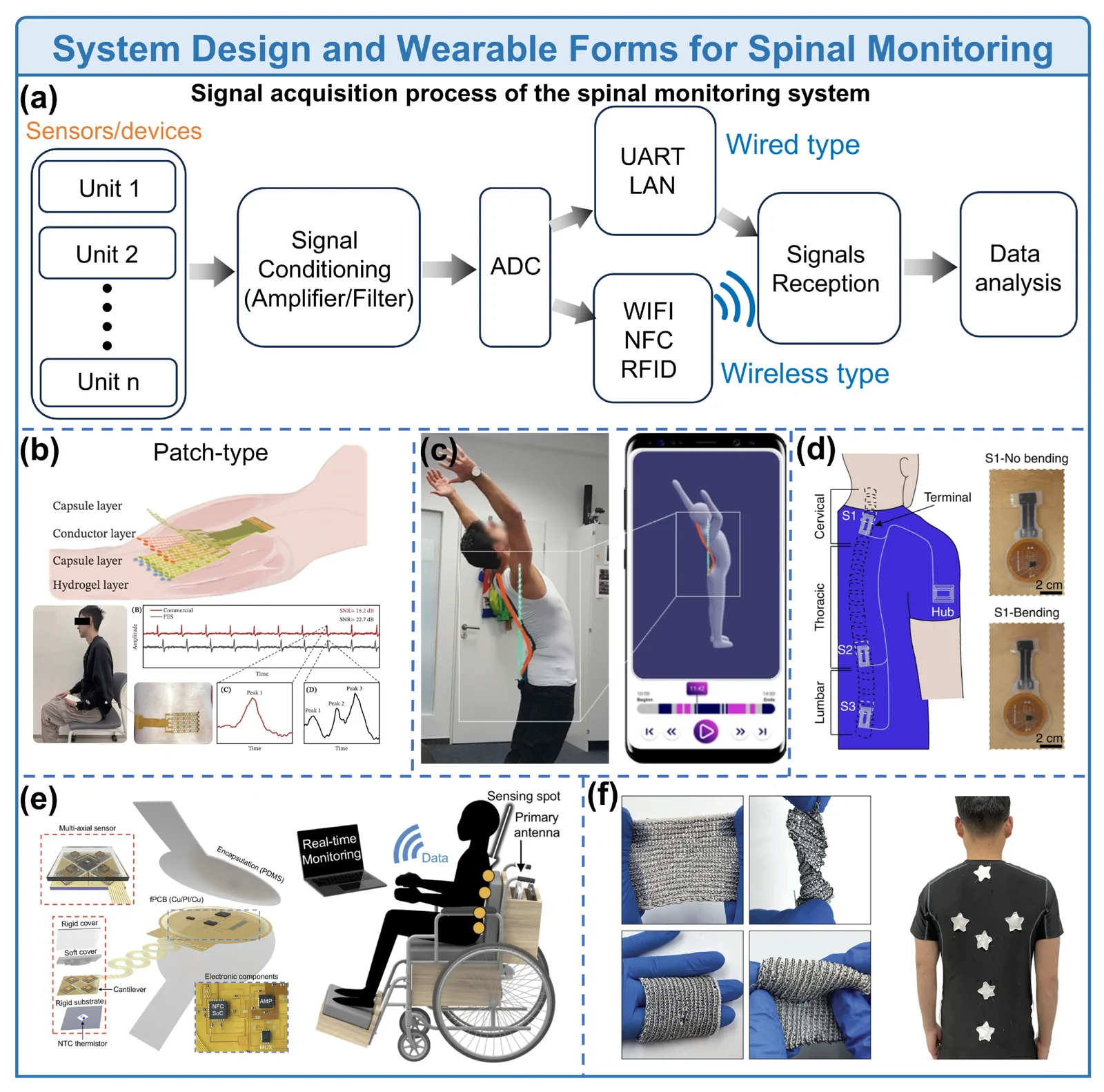
System design and wearable forms for spinal monitoring. (**a**) Typical architecture of wearable spinal monitoring systems, consisting of sensing acquisition, signal conditioning, analog-to-digital conversion, data transmission and intelligent analysis modules. (**b**) Wired hydrogel-based EMG sensing patch for clinical lumbar electrophysiological detection (reprinted from [13], Copyright 2024, with permission by CC BY 4.0). (**c**) Wireless flexible device for 3D spinal posture tracking and dynamic motion data collection (reprinted from [31], Copyright 2023, with permission by CC BY 4.0). (**d**) Near-field enabled clothing with conductive fiber embroidery for battery-less distributed sensing (reprinted from [32], Copyright 2024, with permission by CC BY 4.0). (**e**) Wireless battery-free multi-axis sensor and near-field clothing for AR-assisted spinal parameter monitoring (reprinted from [33], Copyright 2025, with permission from Springer Nature). (**f**) Triboelectric self-powered sensing vest for daily sitting posture monitoring with high recognition accuracy (reprinted from [38], Copyright 2022, with permission from Springer Nature).

The design of wearable spinal monitoring systems follows a standardized process of sensing acquisition–signal conditioning–analog-to-digital conversion–data transmission–intelligent analysis. As shown in Figure 3a, after unitary or multi-unit sensing modules collect spinal motion and physiological signals, the signal-to-noise ratio (SNR) is optimized through signal conditioning steps such as amplification and filtering, followed by analog signal digitization via an analog-to-digital converter module. Finally, data is transmitted to the terminal for analysis through wired (UART/LAN) or wireless (WiFi/NFC/RFID) means. The entire architecture generally adheres to the core principles of modularization, low power consumption, and high stability, adapting to the complex physiological structure and dynamic motion characteristics of the spine, and laying a fundamental framework for the technological iteration of different wearable forms.

Wired patch-type systems were the early mainstream form of wearable spinal monitoring technology, and have become a core solution for clinical accurate detection by virtue of high sensing accuracy and signal stability. As shown in Figure 3b, the flexible electromyographic sensing patch based on in-situ hydrogel proposed by Mingxuan Zhang et al. takes thermoresponsive hydrogel and flexible grid electrodes as the core [13], achieving high adhesion to the skin through temperature-triggered sol–gel transition of the hydrogel. This effectively reduces contact impedance and improves the SNR of electromyographic signals, enabling accurate capture of subtle electrophysiological signals in the early stage of lumbar diseases and providing a basis for the early quantitative diagnosis of spinal diseases. The advantages of such wired patch-type systems include low data transmission delay and small signal interference, making them suitable for local accurate monitoring in clinical scenarios. However, the inherent wired design limits the user’s activity freedom, failing to meet the needs of long-term home rehabilitation and daily dynamic monitoring. For this reason, many research teams have begun to explore wireless communication methods. For example, the research by Mostafa Haghi et al. adopted FlexTail^®^ (Minktec, Braunschwieg, Lower Saxony, Germany), a flexible wearable device for three-dimensional spinal posture tracking composed of strain gauges and a bottom acquisition circuit (Figure 3c) [31]. This device is placed at the spinal curvature of the subject to record motion data such as spinal tilt changes, and completes daily activity classification and real-time posture tracking combined with deep learning algorithms such as LSTM and CNN. The application of data fusion and principal component analysis further improves the recognition accuracy of spinal bending movements, demonstrating the technical potential of wireless patches in dynamic monitoring.

With the increasing demand for portability and dynamic monitoring, wireless patch-type systems have further optimized skin-friendliness and functionality. The skin-integrated cervical posture monitoring device proposed by Hu Luo et al. integrates flexible electronic circuits and tactile feedback modules into electronic skin through a tether-free design [24]. It uses an accelerometer to sense changes in cervical posture and a vibration actuator to provide four-way tactile correction signals. Meanwhile, a hollow structure design ensures skin attachment stability and sensing reliability during cervical movement. Inheriting the high skin-friendliness and deployment flexibility of patch-type systems, such wireless patch-type systems solve the portability problem of wired design through wireless communication, balancing detection accuracy and dynamic activity adaptability, and are suitable for real-time monitoring of local areas such as the cervical and lumbar spine. However, the monitoring range of a single patch is limited, making it difficult to achieve synchronous acquisition of multi-parameters for the entire spinal region. Additionally, technical challenges such as data synchronization and power consumption management exist in multi-node deployment.

To meet the demands of global distributed monitoring and long-term home rehabilitation, wearable forms are evolving toward textile-integrated systems, simultaneously realizing a leap from single-point sensing to networked monitoring. Rongzhou Lin et al. developed near-field enabled clothing and a wireless body sensor network [32], which seamlessly integrate near-field response patterns into fabrics through computer-controlled conductive fiber embroidery technology (Figure 3d), achieving wireless power supply and data connection at multiple distal points of the body and constructing a battery-less distributed sensing network. In addition, the integration system of wireless battery-less multi-axis sensors and near-field enabled clothing proposed by Hyeonseok Han et al. (Figure 3e) combines miniaturized battery-less sensors with textile substrates [33], realizing wireless power supply and data transmission through near-field communication, and enabling visual monitoring of spinal pressure, temperature and other parameters in combination with an augmented reality system, which exhibits significant application value in spinal posture tracking and intervertebral disc herniation prevention. Furthermore, the Straighten Up + device proposed by Gabriela Cajamarca et al. optimizes its adaptability for the elderly on the basis of textile-type systems [34]. Integrated with a multi-dimensional accelerometer and an adjustable strap vest, it achieves a 93.5% recognition accuracy for eight daily postures of the elderly, while taking into account the user experience and acceptance among elderly users, providing a practical reference for long-term spinal posture monitoring of special populations.

Junyeong Lee et al. proposed an all-textile, chip-free, and battery-less human body sensor network [35], which relies on a concentric multi-node hub antenna architecture and flexible conductive fiber embroidery technology to realize single-frequency scanning synchronous monitoring of whole-body distributed passive sensors, further expanding the global monitoring capability of textile-type systems. Moreover, the integration system of an inter-implant wireless network and metamaterial textiles proposed by Xi Tian et al. extends the application boundary of textile-type sensing networks to the field of implantable bioelectronics [36]. It uses metamaterial textiles to realize non-radiative propagation of radio frequency signals along the body surface and passively amplify the received signal strength by more than three orders of magnitude, providing a brand-new solution for the integrated in vivo and in vitro monitoring of postoperative spinal rehabilitation. Also based on metamaterial-based integrated design, the textile-integrated metamaterial scheme proposed by Amirhossein Hajiaghajani et al. realizes long-distance battery-less communication between NFC devices through an array design of anisotropic magnetic sensing elements [37], promoting the development of textile-type systems toward multi-device and cross-subject interconnection.

For sedentary populations and special rehabilitation scenarios, Yang Jiang et al. developed a self-powered sitting posture monitoring vest based on triboelectric nanogenerators (Figure 3f) [38]. It interweaves conductive fibers with nylon yarn through a knitting double-thread process and integrates an array of triboelectric sensors to form a self-powered monitoring vest, which features high sensitivity, good elasticity and wash stability. Combined with a random forest classifier, it achieves a 96.6% sitting posture recognition accuracy, adapting well to daily spinal health monitoring. Zhipeng Li et al. proposed a body sensor network based on flexible topological clothing, which replaces the traditional one-dimensional unit structure with a two-dimensional topological module [39], greatly improving signal transmission efficiency and performance stability under bending conditions. Combined with machine learning algorithms, it achieves high-precision monitoring of spinal-related vital signs, with a SNR improved by more than two orders of magnitude compared with traditional networks, further expanding the technical boundary and application potential of textile-type systems.

Overall, three types of wearable forms, wired patch, wireless patch, and textile-integrated, constitute the core technical systems of soft sensing systems for spinal monitoring, and they form a distinct complementarity in terms of performance and scenario adaptability: Wired patch-type systems have high accuracy and high stability as core advantages, adapting to clinical accurate local detection. Wireless patch-type systems balance detection accuracy and dynamic portability, making them suitable for real-time dynamic monitoring of local spinal regions, while textile-integrated systems have become the mainstream solution for daily prevention and long-term home rehabilitation monitoring by virtue of global distributed monitoring, high wearing comfort and wireless networking capability. From the perspective of technological evolution logic, the design of wearable spinal monitoring systems has always centered on the core demands of non-invasiveness, continuity, quantifiability and home compatibility. Wirelessization, battery-less operation, self-power supply and intelligent analysis have become core development trends. The in-depth fusion of technologies such as metamaterial textiles, triboelectric self-power supply, near-field communication, and implant–surface interconnection continuously drives the system toward high integration, high stability and multi-scenario adaptability. In the future, its research and development will further focus on the customized adaptation of clinical and daily scenarios. The integrated design of sensing–transmission–analysis–intervention will promote the large-scale clinical implementation of flexible wearable technology in the prevention, diagnosis and rehabilitation of spinal diseases, providing a new technical paradigm for spinal health management.

## 5. Signal Processing and Core Algorithms Analysis

**Figure 4:**
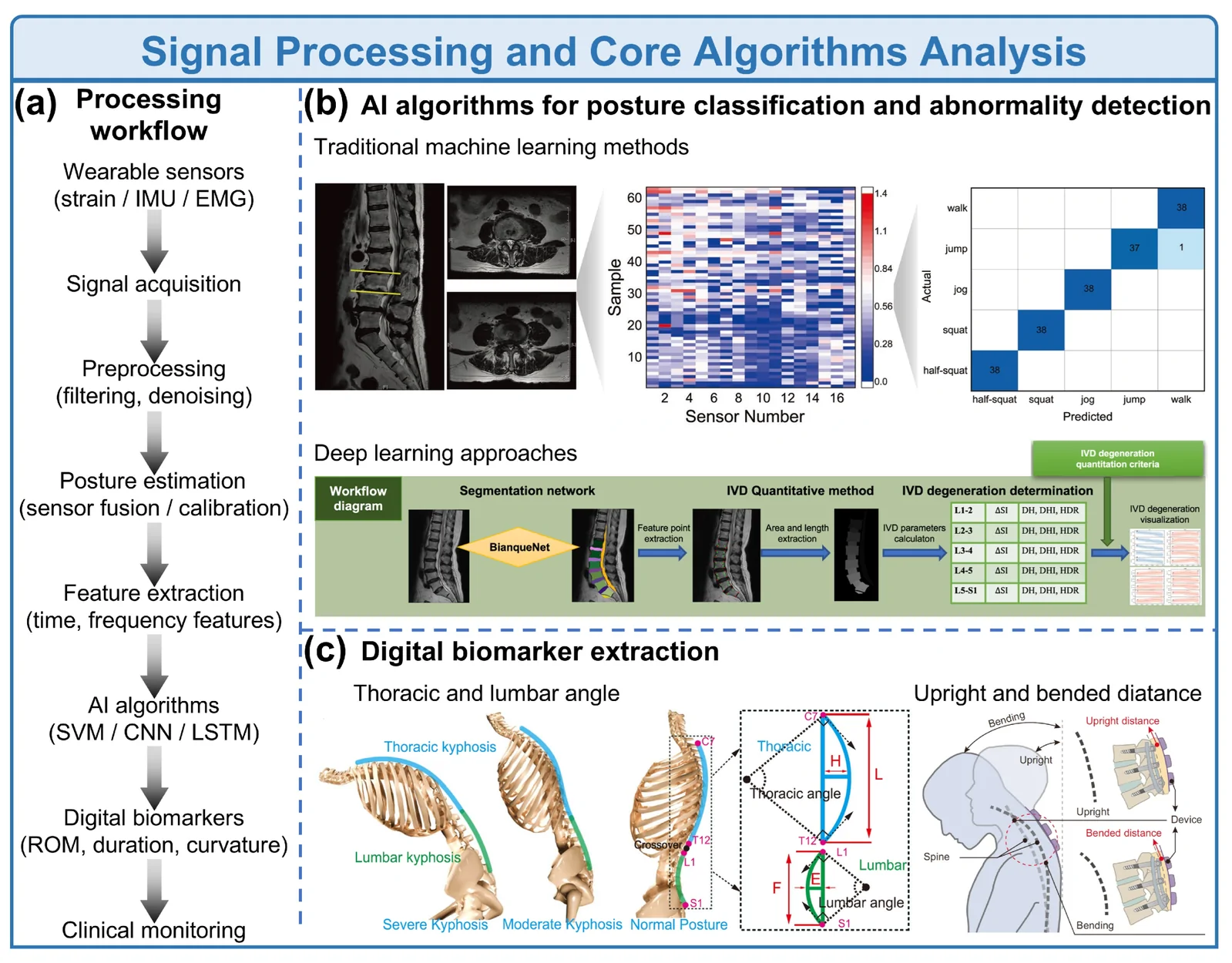
Signal processing and core algorithms analysis. (**a**) Signal processing pipeline. Data from wearable sensors (strain, IMU, EMG) undergoes preprocessing (filtering, denoising), posture estimation (sensor fusion, calibration), feature extraction (time- and frequency-domain attributes), and AI algorithms (SVM, CNN, LSTM) to generate digital biomarkers (ROM, posture duration, curvature) for clinical monitoring. (**b**) Algorithm comparison of traditional machine learning methods and deep learning approaches in the algorithm analysis (reprinted from [52]. Copyright 2022, with permission by Wiley-VCH). (**c**) Digital biomarker extraction based on thoracic and lumbar angles (reprinted from [53]. Copyright 2021, with permission by Springer Nature) as well as upright and bended distance (reprinted from [20]. Copyright 2024, with permission by Springer Nature).

Wearable soft sensors generate continuous biomechanical signals that reflect spinal posture, motion patterns, and muscle activity. However, raw signals are often contaminated by motion artifacts, sensor drift, and environmental noise. Therefore, robust signal processing and data-driven algorithms are essential for extracting clinically meaningful information. A typical analysis pipeline includes signal preprocessing for posture estimation, machine-learning-based classification, and digital biomarker extraction [40,41,42,43] (Figure 4).

The first step in wearable spine monitoring is signal preprocessing, which improves signal quality and removes noise from sensor measurements. Flexible strain sensors, IMUs (Inertial Measurement Units), and EMG sensors generate high-frequency time-series signals that typically require filtering before further analysis. Common approaches include low-pass filtering, band-pass filtering, and wavelet denoising (Figure 4a), which suppress motion artifacts while preserving physiologically meaningful information [43,44]. After preprocessing, the system proceeds to posture estimation or spinal kinematic reconstruction. In IMU-based systems, spinal orientation is typically obtained via sensor fusion algorithms that integrate accelerometer and gyroscope measurements. Within the framework of rigid-body kinematics, orientation is defined as the rotation of a local coordinate frame with respect to a global reference frame. Let $q_{\mathrm{tor}}^{G}$ and $q_{\mathrm{arm}}^{G}$ denote the orientations of the torso and arm frames relative to a common global frame *G*, respectively. The shoulder orientation *q*_sh_, defined as the orientation of the arm frame with respect to the torso frame, can then be expressed as:

(1) $$q_{\mathrm{sh}}={\left(q_{\mathrm{tor}}^{G}\right)}^{*}q_{\mathrm{arm}}^{G}$$

where (•)^∗^ denotes quaternion conjugation. To compensate for inertial drift, a drift extraction module is introduced, which estimates IMU drift by fusing IMU-derived orientations with the shoulder orientation obtained from soft sensors. Specifically, the drift quaternion *q*_drift_ is defined as:

(2) qdrift=qtor,IMU qsh,soft sensorsqarm,IMU∗

where *q*_tor,IMU_ and *q*_arm,IMU_ represent the raw orientation measurements from the torso and arm IMUs, respectively. To extract the physically meaningful drift component, it is assumed that IMU drift predominantly manifests in the yaw direction. Accordingly, the yaw angle *ψ* is extracted from *q*_drift_ as:

(3) $$\psi =\arctan\left(\frac{2\left(\mathrm{wz}+\mathrm{xy}\right)}{1-2\left(y^{2}+z^{2}\right)}\right)$$

where *w* is the scalar component and *x*, *y*, *z* are the vector components of *q*_drift_ [45,46]. For strain-based wearable soft sensors, electrical signals such as resistance or capacitance variations are used to quantify spinal deformation. These sensing mechanisms enable real-time monitoring of spinal curvature and motion during daily activities, including sitting, bending, and walking [25,47]. Following posture estimation, signals are segmented into time windows for feature extraction. Typical features include statistical metrics (mean, variance, *p*-value, etc.), temporal characteristics (peak amplitude, duration), and frequency-domain parameters obtained via Fourier or wavelet transforms. These extracted features are then used as inputs for machine-learning models designed for posture classification and abnormal movement detection [48].

Machine learning algorithms have significantly enhanced the capability of wearable systems to interpret complex motion signals. In spine monitoring applications, AI models are primarily used for posture classification, movement recognition, and abnormal posture detection (Figure 4b). Traditional machine learning methods such as support vector machines (SVM), random forests, and k-nearest neighbors (KNN) are widely used because they perform well on relatively small datasets and provide interpretable classification results (shown in Table 2) [48]. These algorithms typically rely on manually extracted features from wearable sensor signals to identify postures such as upright sitting, forward bending, or slouching.

With the rapid development of wearable computing and data availability, deep learning approaches have become increasingly popular. Convolutional neural networks (CNNs) are capable of automatically extracting spatial–temporal features from sensor data, while recurrent neural networks (RNNs), especially long short-term memory (LSTM) networks, effectively capture temporal dependencies in motion signals. These architectures have demonstrated high accuracy in human activity recognition and wearable motion monitoring tasks [42,49]. Recent studies have also demonstrated real-time wearable motion tracking systems with deep learning-based classifiers that are capable of recognizing subtle human movement patterns with millisecond-level latency [42]. As summarized in Table 2, such systems can detect prolonged poor posture, asymmetric spinal loading, or abnormal movement patterns associated with musculoskeletal disorders, enabling real-time feedback for posture correction and rehabilitation training.

Beyond posture classification, wearable sensor systems enable the extraction of digital biomarkers (Figure 4c), which transform raw sensor data into clinically interpretable indicators of health status. Digital biomarkers are commonly defined as objective, quantifiable physiological and behavioral measurements collected through digital devices such as wearable sensors or mobile technologies that can explain, influence, or predict health outcomes [50]. Compared with traditional clinical measurements, digital biomarkers enable continuous, high-frequency, and real-world monitoring of physiological functions, allowing clinicians to capture long-term disease progression and treatment responses [51].

In spine monitoring applications, typical digital biomarkers like spinal curvature angle (cervical, thoracic, lumbar), range of motion (ROM) during flexion, extension, and rotation, posture stability index (reflecting trunk motion variability and sedentary posture duration or indicating prolonged sitting or slouching, and movement symmetry) are useful for rehabilitation evaluation. Additionally, multimodal sensing has emerged as a powerful strategy for comprehensive spine monitoring, enabling more robust state analysis by integrating heterogeneous physiological and biomechanical signals [54]. Compared with single-modality sensing, multimodal fusion improves robustness against motion artifacts and enhances the accuracy of posture estimation and abnormality detection. Data processing typically involves temporal alignment, normalization, and feature-level or decision-level fusion, followed by machine learning or deep learning models for pattern recognition. Furthermore, multimodal data facilitate the extraction of richer digital biomarkers, such as coordinated motion–muscle coupling indices and dynamic stability metrics [55], which are difficult to derive from a single signal source. This integrated approach provides a more holistic representation of spinal health and holds significant potential for improving early diagnosis, rehabilitation monitoring, and personalized therapeutic interventions.

These metrics provide quantitative indicators for evaluating spinal disorders such as LBP, scoliosis, and post-operative recovery. Wearable sensor systems also enable remote monitoring in home environments, allowing clinicians to observe patient behavior outside traditional clinical settings and facilitating personalized rehabilitation strategies [52,56]. Future research is expected to integrate multimodal sensing, including biomechanical signals, muscle activity, and physiological parameters [57,58], combined with advanced machine learning techniques to construct comprehensive digital phenotypes of spinal health. Such approaches may significantly improve early diagnosis and personalized management of spinal disorders [59,60].

## 6. Clinical and Translational Evidence

Wearable soft sensors have emerged as a promising technology for monitoring spinal biomechanics in real-world environments. Unlike conventional imaging-based diagnosis (e.g., X-ray or MRI), wearable systems enable continuous, non-invasive, and quantitative monitoring of spinal posture and motion during daily activities [26]. However, the evidence supporting these systems varies substantially in terms of what is measured and how performance is validated. Analytical or bench-level performance, such as sensor resolution, sensitivity, or signal-to-noise ratio (SNR), controlled posture classification, testing in healthy volunteers, patient-based validation, and routine clinical implementation represent distinct stages of evidence and should not be treated as equivalent. Recent advances in flexible electronics, fiber-based strain sensors, and inertial sensing platforms have enabled real-time measurement of spinal curvature, trunk motion, and paraspinal muscle activity, providing a new paradigm for spinal disease management and rehabilitation [61,62,63]. The core measurement principle of most wearable spine sensors is based on mechanotransduction, where mechanical deformation of a flexible sensor is converted into electrical signals [64]. By integrating these sensors into smart garments, orthotic braces, or skin-conformal patches, wearable systems can continuously monitor spinal alignment, provide real-time feedback, and support long-term clinical management.

**Figure 5:**
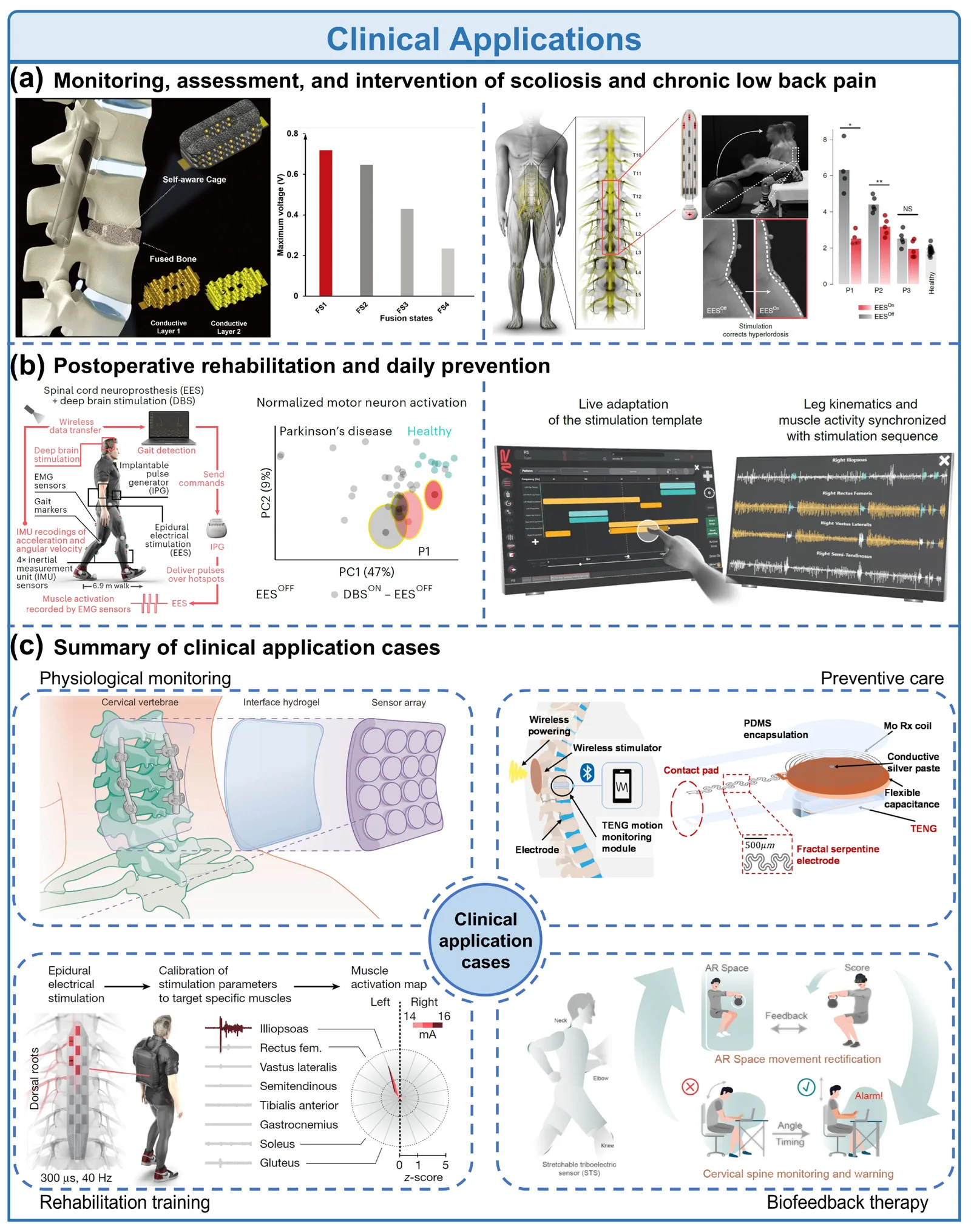
Clinical applications. (**a**) Continuous monitoring and feedback-enabled assessment of spinal curvature and motion for scoliosis (reprinted from [16]. Copyright 2022, with permission by Wiley-VCH) and chronic LBP (reprinted from [26]. Copyright 2022, with permission by Springer Nature). (Mann–Whitney test, two tailed: * *p* < 0.05; ** *p* < 0.01). (**b**) Real-time tracking of spinal movement and posture to support postoperative rehabilitation (reprinted from [14]. Copyright 2023, with permission by Springer Nature) and daily prevention (reprinted from [26]. Copyright 2022, with permission by Springer Nature). (**c**) Integrated clinical framework covering physiological monitoring (reprinted from [20]. Copyright 2024, with permission by Springer Nature), rehabilitation training (reprinted from [80] Copyright 2024, with permission by Springer Nature), biofeedback therapy (reprinted from [84]. Copyright 2025, with permission by Wiley-VCH), and preventive care (reprinted from [64]. Copyright 2025, with permission by Elsevier).

Spinal disorders such as adolescent idiopathic scoliosis (AIS) and chronic LBP represent major global health challenges. Epidemiological studies indicate that AIS affects approximately 2–4% of adolescents, while chronic LBP is one of the leading causes of disability worldwide [65,66]. Conventional diagnosis relies primarily on radiographic measurements such as the Cobb angle, defined as the angle between the upper and lower endplates of the most tilted vertebrae in a spinal curve [67,68,69]. Although effective for structural assessment, it provides only static snapshots, exposes patients to repeated radiation, and cannot capture dynamic postural behaviors during daily activities. Wearable soft sensors offer a complementary solution by enabling continuous monitoring of spinal kinematics [70] (Figure 5a). For instance, flexible strain sensors arranged along the back can measure local curvature changes associated with spinal deformation [71]. By correlating the measured strain with spinal curvature, the system can estimate spinal alignment in real time. This highlights the growing demand for smart medical devices that integrate advanced functionalities. Several wearable systems have been developed for scoliosis monitoring [72]. Sensor-integrated orthotic braces equipped with pressure and curvature sensors can track brace compliance and corrective force distribution during treatment [73]. Clinical studies have shown that wearable monitoring platforms can significantly improve patient adherence to brace therapy and enable remote monitoring of treatment progress [73,74]. In response, the proposed self-aware implants leverage metamaterial design and nano energy harvesting to achieve self-sensing, self-powering, and mechanical tunability, offering a transformative approach for next-generation implantable systems [16]. For chronic LBP, wearable systems provide valuable information about lumbar motion patterns and postural loading [75,76]. Sensors such as IMUs, flexible bending sensors, and sEMG electrodes can be combined to monitor trunk angles, muscle activity, and movement symmetry. Abnormal movement patterns, such as excessive lumbar flexion or prolonged static posture, are strongly associated with increased spinal loading and pain risk [26]. Recent wearable systems integrating curvature sensors and sEMG electrodes have demonstrated the ability to identify asymmetric paraspinal muscle activity and predict spinal curvature direction with accuracies above 90%, indicating strong potential for early detection of postural abnormalities [77]. However, such results should be distinguished from validated disease-screening or diagnostic performance, because their relationship with pain severity, functional limitation, disease progression, and treatment response requires prospective clinical validation. Wearable devices can provide real-time biofeedback through vibrotactile or smartphone-based alerts, encouraging users to correct poor posture or perform rehabilitation exercises [78]. In addition, wearable biofeedback systems can support sensorimotor retraining, a rehabilitation strategy aimed at restoring proper spinal motor control. These systems deliver immediate feedback when abnormal postures or movement patterns are detected, thereby promoting motor learning and improving spinal stability [67]. Such approaches are increasingly integrated into digital rehabilitation platforms and telemedicine systems.

Representative studies illustrate the distinction between technical measurement performance and clinical evidence. Bartalesi et al. combined textile strain sensors with accelerometers to estimate lumbar curvature and reported a 2% error in lumbar-arch length reconstruction against stereophotogrammetry in preliminary testing [12]. This result supports the feasibility of wearable lumbar-curvature measurement but does not by itself establish diagnostic performance in patients. Similarly, a skin-integrated cervical sensing device was evaluated against a digital level and subsequently tested in task-based studies involving 12 healthy graduate students; the reported pitch and roll root-mean-square errors were 1.37° and 1.14°, respectively [24]. These results provide evidence of measurement feasibility and short-duration usability in healthy volunteers, but they should not be interpreted as evidence for disease screening or treatment efficacy. The FlexTail system further demonstrated three-dimensional spinal-pose tracking during activities of daily living in 30 healthy adults [31]. Although this study involved a larger healthy-volunteer dataset and therefore provides useful evidence for real-world motion measurement, it still does not constitute patient-based clinical validation.

Beyond disease monitoring, wearable soft sensors play an increasingly important role in postoperative rehabilitation and preventive healthcare [44] (Figure 5b). Patients undergoing spinal surgeries, such as spinal fusion, discectomy, or deformity correction, require long-term monitoring of spinal motion and rehabilitation progress. However, conventional follow-up assessments typically rely on periodic clinical visits and imaging examinations, which provide limited information about functional recovery between visits. Wearable systems enable continuous tracking of spinal ROM and daily physical activity, offering valuable information for evaluating postoperative recovery [79]. Range of motion can be calculated using orientation data from IMU sensors based on Euler angles [80]. Using these measurements, clinicians can quantify lumbar flexion, extension, and rotation during rehabilitation exercises. If movements exceed predefined safety thresholds, wearable systems can provide immediate feedback to prevent excessive loading on healing tissues.

Recent developments in smart textile systems further enhance wearable monitoring capabilities. Fiber-based strain sensors and conductive textile networks can be seamlessly integrated into clothing, enabling large-area motion sensing while maintaining comfort and breathability [38,71]. Such systems allow long-term monitoring of spinal posture during everyday activities, including sitting, standing, and walking. For healthy individuals, wearable posture-monitoring devices can serve as preventive tools against spinal degeneration. Poor posture, such as forward head posture or prolonged slouching, can significantly increase intervertebral disc pressure and accelerate musculoskeletal degeneration. Wearable posture correction devices use real-time feedback mechanisms to promote proper alignment and reduce cumulative spinal loading [38,81]. In occupational settings, wearable systems have also been applied to monitor spinal strain during manual labor tasks [26]. Smart textile-based exosuits and sensor-integrated garments can detect excessive bending or lifting loads and assist the erector spinae muscles, reducing musculoskeletal injury risk. Furthermore, the integration of AI and machine learning enables more advanced analysis of wearable sensor data [82]. Machine-learning algorithms can extract motion patterns from complex datasets, enabling accurate classification of spinal movements and prediction of injury risks [83]. These data-driven approaches may eventually support personalized rehabilitation programs and earlier identification of abnormal movement patterns, but their clinical utility depends on external validation and demonstrated improvement in clinically meaningful outcomes.

In recent years, multiple clinical studies have demonstrated the feasibility and clinical value of wearable spine-monitoring technologies. Current applications can be broadly categorized into three primary clinical scenarios (Figure 5c). First, wearable sensors are widely used for posture and spinal curvature monitoring. Flexible strain sensors, IMUs, and pressure sensors can capture real-time spinal alignment data, enabling continuous assessment of posture and movement in daily life [26]. These systems provide a radiation-free alternative for monitoring scoliosis progression and evaluating treatment outcomes. Second, wearable devices have been successfully applied in rehabilitation training and biofeedback therapy. By providing real-time feedback on posture and movement quality, these systems improve patient engagement and adherence to rehabilitation protocols [80]. Clinical studies have shown that wearable biofeedback can enhance proprioception and improve spinal motor control in patients with chronic LBP [83,84]. Third, wearable systems enable long-term health monitoring and preventive care. Continuous data collection provides valuable insights into habitual movement patterns, occupational risk factors, and lifestyle-related spinal stress [64]. Table 3 summarizes representative studies according to system type, study population and setting, comparator, reported outcome, and validation level. Sample sizes are stated when reported in the cited studies. Where a source does not provide a clinical cohort, the corresponding study is identified as bench testing, controlled-task evaluation, healthy-volunteer validation, or proof-of-concept evidence rather than being classified as clinical validation. These datasets can support predictive modeling and personalized healthcare strategies.

Despite significant progress, several challenges remain. Clinical validation studies are still limited in scale, and standardization of sensor placement, calibration methods, and data interpretation is needed. Furthermore, ensuring long-term comfort, power efficiency, and data security remains essential for widespread clinical adoption. Overall, wearable soft sensors have the potential to complement conventional imaging and clinical examination by transforming spinal monitoring from an episodic measurement paradigm toward more continuous observation of posture, motion, interface force, and muscle activity. However, the current literature supports this technology primarily as an emerging measurement and monitoring platform rather than as a fully established diagnostic or therapeutic system. With continued advances in flexible electronics, multimodal sensing, artificial intelligence, and telemedicine infrastructure, future research should emphasize not only higher sensing performance but also standardized validation, longitudinal clinical studies, and demonstrated clinical utility. Such evidence will be essential for translating wearable spinal-monitoring technologies from laboratory prototypes and controlled volunteer studies into reliable tools for routine clinical care.

## 7. Challenges & Future Directions

The development of current wearable soft sensors for spinal monitoring still faces two core bottlenecks. In terms of long-term stability, soft sensing materials are susceptible to human sweat, body temperature and repeated mechanical deformation, leading to signal drift, sensitivity attenuation, and calibration changes, while sensor displacement, garment fit, and skin–sensor contact can further affect signal quality. These factors make it difficult to maintain reliable performance during long-term continuous monitoring, particularly during the rehabilitation stage of spinal diseases. In terms of clinical adaptability, most existing devices are developed based on laboratory scenarios and lack customized designs for different diseases such as scoliosis and chronic LBP. In addition, sensor placement, calibration procedures, reference methods, and data-processing pipelines have not been sufficiently standardized across studies, limiting comparison and replication. The clinical thresholds for interpreting wearable monitoring data also remain insufficiently established, resulting in limited connection with established clinical diagnosis and treatment systems. Meanwhile, the balance between wearing comfort, monitoring accuracy, power management, and multimodal data synchronization remains important for clinical translation. Patient cohorts, longitudinal adherence, external validation, and evidence of clinical utility are still limited, while data governance and equitable usability require further attention before widespread deployment.

In the future, wearable soft sensing technology for spinal monitoring will develop around three core directions: soft sensing upgrading, tele-rehabilitation implementation, and in-depth clinical integration. At the soft-sensing level, new materials with improved stability, biocompatibility, and mechanical durability, together with heterogeneous integration processes, will be developed to improve sensing reliability under repeated deformation and environmental variation. Future studies should also adopt more standardized reporting of material composition, device dimensions, sensing ranges, hysteresis, drift, repeatability, comparator methods, and failure rates. In the field of tele-rehabilitation, relying on 5G and the Internet of Things technologies will realize real-time transmission and cloud analysis of sensing data, and build a personalized remote posture correction and rehabilitation guidance system combined with AR/VR technologies. However, remote monitoring and feedback should be evaluated for safety, adherence, and clinical benefit before claims of effectiveness are made. At the clinical integration dimension, future research should strengthen the connection between monitoring data and clinical diagnosis and treatment standards, develop customized wearable devices for different spinal diseases, examine the full cycle of spinal health management from early disease screening and intraoperative monitoring to postoperative rehabilitation, and ultimately complete the transformation from laboratory technology to routine clinical diagnosis and treatment tools.

## 8. Conclusions

Wearable soft and hybrid sensing systems provide a promising non-invasive approach for continuous and quantitative monitoring of spinal movement, posture, interface forces, and muscle activity during laboratory tasks and daily activities. Their key advances in compliant materials, diverse sensing mechanisms, wearable integration, multimodal fusion, and intelligent signal processing have expanded the capability of spinal monitoring beyond the episodic structural information provided by conventional imaging. From material development and sensor integration to system-level design and data analysis, these technologies have established a versatile framework for dynamic spinal-health assessment, with encouraging potential in applications such as scoliosis screening, chronic LBP assessment, and postoperative rehabilitation tracking. However, the current evidence remains largely based on bench testing and feasibility studies, including studies in healthy volunteers, and reported improvements in angular error, signal quality, force response, or posture classification should not be interpreted as equivalent to patient-level diagnostic accuracy or improved clinical outcomes. Future progress therefore requires standardized testing and calibration protocols, reproducible performance evaluation, external validation, and adequately designed studies involving relevant patient populations and long-term monitoring. With improved material and system stability, greater clinical adaptability, and appropriately validated remote rehabilitation capabilities, wearable sensing may become a valuable adjunct to clinical assessment and personalized rehabilitation. Nevertheless, its effectiveness and role in routine spinal care remain to be established, and successful clinical translation will depend on evidence-based integration rather than technological capability alone.

## Figures and Tables

**Table 1:** Typical diverse sensing principles sensors for spinal monitoring.

Sensor/Mechanism	Materials or Configuration	Reported Metric or Range	Technical Advantages	Core Limitations
Magnetic	Battery-free implantable magnetic sensing system [11]	Wireless, battery-free spinal-motion monitoring demonstrated in rats and humans;	Wireless and batteryless; implantable enables long-term in vivo monitoring, strong anti-interference	Implantable is invasive; surface type is susceptible to external magnetic field interference, high cost
sEMG	Mesh electrode with in-situ-gelling gelatin/PCA-Na hydrogel [13]	40 kΩ/cm^2^ at 10 Hz; SNR 23.28 dB; hydrogel resistance decreased 82% at 40 wt% PCA-Na vs. gelatin hydrogel	Reflects the coordinated movement state of muscles and spine, assists in judging abnormal posture	Easily affected by skin contact state, requiring complementation with other sensing technologies
Piezoelectric	Flexible PVDF/copolymer or composite structures [15]	Dynamic pressure/force; sensitivity; response time; detectable vibration frequency	Fast response speed, adaptable to dynamic stress monitoring, self-powered	Poor static monitoring performance, sensitivity greatly affected by structural design
Piezoresistive	MXene/sponge dual conductive paths [22]	Sensitivity: 1.3 × 10^6^ kPa^−1^ at low pressure and 1.9 × 10^5^ kPa^−1^ at higher pressure; 100% predefined posture-class accuracy in one dataset	High sensitivity, adaptable to large-deformation monitoring, low cost, easy arrayation	Prone to fatigue drift during long-term use, greatly affected by temperature and humidity
Optical (FBG fiber optic)	Four silicone-embedded FBGs in knitted undergarments [23]	Bench force application up to 10 N on artificial skin;	High detection accuracy, good linearity, anti-electromagnetic interference, resistant to harsh environments	Limited flexible adaptability, slow response in dynamic monitoring, relatively complex equipment
IMU inertial	Accelerometer-based skin-integrated cervical device [24]	Against digital level: pitch RMSE 1.37° and roll RMSE 1.14°; separate 12-person healthy-volunteer study	Mature technology, accurate dynamic monitoring, easy miniaturization and integration, moderate power consumption	Prone to cumulative errors, requiring algorithm calibration, unable to directly detect stress
Capacitive	Flexible electrode/dielectric structures [25]	Trunk flexion and torsion angle estimation error approximately 8°–15	Low power consumption, good response linearity, anti-mechanical fatigue	Easily affected by environmental humidity, high packaging requirements
Triboelectric	Grating-structured retractable sensor [26]; Stretchable array [14]	8 V mm^−1^ sensitivity, 0.6 mm minimum resolution, >120,000 cycles; separate array: ≈1° resolution, 0.56 V °^−1^, 98.7% for 14 postures	Self-powered and low power consumption, high angular resolution, fast response speed	Output signal easily affected by contact state, poor static monitoring performance

**Table 2:** Representative algorithms for wearable spine monitoring.

Algorithm Type	Representative Methods	Key Function	Advantages	Limitations
Signal Filtering	Low-pass filter, Wavelet denoising	Remove noise and motion artifacts	Simple implementation, real-time capability	Limited ability to remove complex artifacts
Sensor Fusion	Complementary filter, Kalman filter	Estimate spinal orientation from IMU signals	Reduces drift and improves accuracy	Requires calibration
Traditional Machine Learning	SVM, Random Forest, KNN	Posture classification and activity recognition	Effective for small datasets, interpretable	Requires manual feature extraction
Deep Learning	CNN, LSTM	Automatic posture recognition and anomaly detection	High accuracy, captures temporal patterns	Requires large datasets and computational resources
Digital Biomarker Modeling	Regression models, feature-based analysis	Quantification of spinal curvature and motion metrics	Clinically interpretable indicators	Standardization still lacking

**Table 3:** Representative clinical and translational evidence for wearable spinal monitoring.

System/Application	Population and Setting	Comparator or Reference	Reported Outcome	Validation Level
Textile piezoresistive array + IMUs; lumbar curvature [12]	Preliminary human test; sample size not specified in the abstract; preliminary human comparison	Stereophotogrammetry	2% error in lumbar-arch length reconstruction	Preliminary technical feasibility; sample size not reported here and no patient cohort
FBG knitted undergarment; brace-interface force [23]	Artificial skin; force applied up to 10 N	Force gauge/calibrated loading setup	Force inferred from Bragg-wavelength shift; patient accuracy not established	Bench validation; no human or patient study
Skin-integrated IMU e-skin; cervical posture and feedback [24]	12 healthy graduate students; three 20-min task scenarios	Digital level for angle sensing; task observations	Pitch/roll RMSE 1.37°/1.14°; feedback feasibility	Bench calibration plus small healthy-volunteer study; no patient cohort
FlexTail flexible strain system; 3D spine pose [31]	30 healthy adults in controlled activity study	Reference labels as described by study	Activity recognition and 3D pose tracking demonstrated	Healthy-volunteer study; not clinical validation
Hydrogel EMG electrode; lumbar muscle signal quality [13]	Human-signal demonstration during sitting; disease cohort not established in summarized report	Commercial electrode/signal and interface measurements	40 kΩ/cm^2^ at 10 Hz; SNR 23.28 dB	Engineering and human-signal feasibility; no validated diagnostic performance
Patient-based validation and routine clinical implementation	No patient cohort or routine-care implementation is identified among these representative studies	Not applicable	No patient-level diagnostic accuracy or outcome benefit established by the studies summarized	Evidence gap: requires prospective patient studies and clinical-utility evaluation

## Data Availability

No new data were created or analyzed in this study. Data sharing is not applicable to this article.
